# Combining Google Earth and GIS mapping technologies in a dengue surveillance system for developing countries

**DOI:** 10.1186/1476-072X-8-49

**Published:** 2009-07-23

**Authors:** Aileen Y Chang, Maria E Parrales, Javier Jimenez, Magdalena E Sobieszczyk, Scott M Hammer, David J Copenhaver, Rajan P Kulkarni

**Affiliations:** 1 Department of Vector-Borne Disease, Nicaraguan Ministry of Health, Managua, Nicaragua; 2 Department of Infectious Disease, Columbia University, New York, NY, USA; 3 Dengue Relief Foundation, Managua, Nicaragua; 4 Department of Vector-Borne Disease, Nicaraguan Ministry of Health, Bluefields, Nicaragua

## Abstract

**Background:**

Dengue fever is a mosquito-borne illness that places significant burden on tropical developing countries with unplanned urbanization. A surveillance system using Google Earth and GIS mapping technologies was developed in Nicaragua as a management tool.

**Methods and Results:**

Satellite imagery of the town of Bluefields, Nicaragua captured from Google Earth was used to create a base-map in ArcGIS 9. Indices of larval infestation, locations of tire dumps, cemeteries, large areas of standing water, etc. that may act as larval development sites, and locations of the homes of dengue cases collected during routine epidemiologic surveying were overlaid onto this map. Visual imagery of the location of dengue cases, larval infestation, and locations of potential larval development sites were used by dengue control specialists to prioritize specific neighborhoods for targeted control interventions.

**Conclusion:**

This dengue surveillance program allows public health workers in resource-limited settings to accurately identify areas with high indices of mosquito infestation and interpret the spatial relationship of these areas with potential larval development sites such as garbage piles and large pools of standing water. As a result, it is possible to prioritize control strategies and to target interventions to highest risk areas in order to eliminate the likely origin of the mosquito vector. This program is well-suited for resource-limited settings since it utilizes readily available technologies that do not rely on Internet access for daily use and can easily be implemented in many developing countries for very little cost.

## Background

Dengue and dengue hemorrhagic fever (DF/DHF) cause significant morbidity and mortality in tropical urban areas. Epidemics of this mosquito borne illness are on the rise worldwide due to increased international travel and unplanned urbanization combined with lack of effective mosquito control measures in tropical developing countries. According to Gubler, there are an estimated 50–100 million dengue infections annually, 5 million DHF cases requiring hospitalization, and 20–25,000 deaths (mainly in children) [1]. As there is currently no effective vaccine for the virus, control measures rely heavily on vector reduction.

In Nicaragua, vector control begins at the municipality level where public health workers from the Ministry of Health survey homes to calculate the mosquito infestation level per neighborhood and identify sites of potential larval development. Mosquito breeding sites can arise in any site with standing water and commonly occur in areas without running water because people often store large uncovered barrels of water around their homes for bathing and drinking. In addition to areas around the home, communal areas such as churches and schools also house large barrels of standing water that lend themselves to larval development and these areas of community gathering can lead to increased transmission of the virus. Standing water is also commonly found in abandoned properties, garbage dumps, and cemeteries where cement flower vases are routine fixtures for graves. Recent studies on the dispersal distance of *Aedes aegypti *indicate that the mosquito generally travels a short distance from its site of release, especially when released indoors or in a dense environment [2]. Mosquitoes released outdoors (with few structures nearby) generally traveled less than 100 m from the release site with the majority recaptured within 60 m, while those released indoors were almost always recaptured within 50 m of the release site [2]. For Bluefields, due to the density of houses and potential larval breeding areas, vector control is focused within a 50 m radius of homes of dengue patients and other hot spots, as the vast majority of mosquitoes are likely to be found at this radius from an identified breeding area.

Nicaragua, like many other developing countries, must deal with the burden of unplanned urbanization. This lack of urban planning has resulted in cities without dependable electricity, running water, sewage systems, paved streets, or a reliable address system. All of these factors represent significant challenges for efficient control of vector-borne disease. The lack of street names and house numbers, for example, makes it difficult to accurately and precisely localize vulnerable sites and deploy necessary resources in an outbreak situation. In this situation, tools for geo-referencing locations by satellite may prove useful because they permit observers at the administrative level to analyze spatial relationships between areas with high levels of mosquito infestation and the locations of sites with a predilection for larval development such as garbage dumps or cemeteries. Resources can then be allocated to perform targeted clean-up of mosquito breeding sites found within garbage piles on an abandoned lot, rather than carrying out widespread fumigation of an entire neighborhood, which has been shown to be ineffective in the control of dengue [3-5]. In addition, it has been shown that the use of geo-referencing saves time in identifying locations when directing different groups of public health workers to return to problem areas to perform control interventions [6].

Though satellite imagery has been available for many years, its uptake had been limited due to cost and quality issues, particularly for public health officials in developing countries [7]. The advent of new mapping technologies such as Google Earth that offers free satellite imagery and aerial photos of most of earth's land surface has lead to the increased uptake of mapping technology for use relevant to health [8-10]. The quality and resolution of the maps offered varies greatly, with better quality maps generally available for Europe and North America, and with cloud cover or shadows sometimes obscuring ground features [9]. However, the resolution is still adequate to identify individual homes in urban areas of most developing nations, including Nicaragua, and thus the Google Earth maps are of sufficient resolution for identifying dengue cases and likely sites of larval development to a 50 m radius or better.

Aside from providing these satellite maps, Google Earth (and Google Earth Pro) has limited map manipulation or analysis functions [11]. ArcGIS 9 ArcMap is a powerful GIS software that is commonly used by public health professionals for generation of geographical data and information. Additionally, ArcGIS is already available to many developing countries participating in the Global Fund for Tuberculosis, Malaria, and AIDS program. Our system incorporates maps from Google Earth into ArcGIS software for further viewing and analysis.

This paper describes the development of a low-cost mapping and georeferencing system which does not rely on continuous access to Internet and was employed first in Bluefields, Nicaragua and then introduced throughout the country as part of a nation-wide initiative. Depending on the resolution of the available satellite imagery, our experience could be replicated in other parts of the world to aid disease surveillance and control efforts, particularly for vector borne disease.

## Methods

### Overview

A base-map was created using the composite of multiple downloaded Google Earth images that was georeferenced. Layers of information were overlain on the base-map such as the locations of potential sites of larval development, the locations of homes of dengue patients, and indices of infestation per neighborhood block to provide administrators at the Ministry of Health with a visual tool to easily identify areas most in need of intervention.

### Creation of the Satellite Base-Map

The Nicaraguan Health System is directed by the Ministry of Health (MINSA) in Managua. Health management is further sub-divided into 17 administrative groups known as SILAIS (Local Systems for the Integral Attention to Health), and each SILAIS is responsible for a group of municipalities (of 152 total). Bluefields is a municipality on the Atlantic coast with 13,174 homes. The municipality of Bluefields was first identified using Google Earth 4.3 (Google, Inc. Mountain View, CA, USA) satellite imagery and the zoom feature was used to create maps with a resolution of 60 cm per pixel; individual homes can be identified at this resolution. This map of Bluefields was then divided into a grid using Google Earth grid overlay function and captured Jpeg images of each grid square at this resolution to create a mosaic with snapshots of the entire municipality.

A complete picture of Bluefields was reconstructed in ArcGIS 9 ArcMap software (ESRI, Redlands, CA USA) using the snapshot Jpeg images. Each image was manipulated and centered to ensure that all images were properly aligned to create a fluid composite picture. The spatial relationships among the images were pegged using control points at the perimeter of the complete picture, which were then saved as an Erdas Imagine (.img) format utilizing the rectify function of the software. The purpose of capturing snapshots of the satellite images in Google Earth and then reconstructing the picture in ArcGIS was so that future users could work with the complete satellite map without the necessity for an Internet connection, as a stable connection can be difficult to obtain in Bluefields.

Next, Erdas Imagine 9.3 (Erdas Inc, Atlanta, Georgia, USA) was utilized to further manipulate the reconstructed picture to create a more fluid transition between the component snapshots. This software is not required for production of the map since the mosaic can be fully constructed in ArcMap; however, we found that this program allowed for enhanced quality of transitions between images. The component snapshot images were cropped and organized in a mosaic and their orientations relative to each other were saved in an .img format. At the completion of this step, the satellite map of Bluefields was one composite image.

### Geo-Referencing of the Composite Map

To geo-reference this composite map, we recorded GPS coordinates at each corner of the map using a Garmin Etrex Summit handheld GPS unit (Garmin, Inc., Olathe, Kansas, USA). Four locations, corresponding to the corners, were visually identified on the composite satellite map. We then travelled to each of those locations and collected the geo-referenced coordinates in UTM format using the GPS units with an error of 5–6 m for each point. Internal consistency of the map was ensured by accurately aligning the snapshot images such that georeferencing of the four corners was sufficient to georeference the composite map to an error of 5–6 m. This precision was confirmed as points collected by GPS units coincided with the home visually identified as the patient's home on the base-map. The coordinates were then entered into the Garmin Mapsource program supplied with the GPS units and the subsequent layer of points was saved as a .dxf file. We then converted this .dxf file into a shapefile using ArcGIS. Each geo-referenced point was subsequently aligned to its location on the satellite image, allowing the user to identify the geographic coordinates of a location by simply moving the cursor over that area. The final geo-referenced image was saved as an .img file.

Neighborhoods and individual blocks were delineated within the final geo-referenced satellite image map using the polygon function in ArcGIS. The neighborhood boundaries were determined by the municipality of Bluefields many years ago and previous public health data is available by neighborhood. Each neighborhood or block was represented with a different polygon. The polygons were saved in a separate layer and superimposed over the satellite image map. Within the attributes table of the polygon layer, information regarding each individual neighborhood was recorded, including access and intermittency of running water, population density, and indices of infestation of the *Aedes aegypti *mosquito responsible for transmitting the dengue virus. Each of these categories in the attributes table represents a risk factor for the transmission of dengue.

### Collection of Data Points and Creation of the Final Satellite Map

In addition to the above information collected per neighborhood block, public health workers also marked the locations likely to have standing water that could lead to the development of *Aedes aegypti *larvae such as landfills, small garbage dumps, abandoned homes that could not be visited by public health workers for interventions, homes of suspected dengue cases, cemeteries, and schools.

These locations were marked using two different methods. The first method involved using GPS devices to collect specific geographic coordinates. These coordinates were then compiled in the Mapsource software and saved as a layer of points in .dxf format, which was then converted into a shapefile in ArcGIS that could be superimposed over the satellite image.

The second method involved printing satellite maps of individual neighborhoods in black and white on 8 × 11 inch sheets of paper. Public health workers then noted locations of likely larval development sites on these paper maps using colored pens. We then created a new shapefile using ArcCatalog, which is an ArcGIS program that allows data access and spatial data management tools, and marked points on the computer image map to correspond to the locations identified on the paper maps. These points were then saved as a shapefile layer utilizing the UTM WGS 1984 16N format.

The final base map is a geo-referenced satellite map of Bluefields in ArcGIS with borders delineating the different neighborhoods. New maps can be created by editing the information in this template with locations of larval development sites or other relevant information to aid in decision making.

### Dengue Fever and Dengue Hemorrhagic Fever (DHF) Case Reporting

Cases of dengue and DHF in Nicaragua are first identified clinically by a physician. Dengue is identified by the Ministry of Health as a febrile illness characterized by headache, retro-orbital pain, myalgias, arthralgias, cutaneous eruptions, diarrhea, and a positive tourniquet test. DHF further includes hemorrhagic manifestations such as petechiae, epistaxis, metrorrhagia, and gingivorrhagia with evidence of pleural effusions, ascites, hemoconcentration 20% above normal, and thrombocytopenia with platelet count less than 100,000/m^3 ^[12]. These cases represent suspected cases.

On the seventh day of the illness, blood samples are drawn and sent to Managua where a serologic ELISA is performed. If positive for IgM antibodies to the dengue virus, the case is reported as confirmed [12].

Since obtaining serological confirmation may take weeks, physicians fill out a form that includes the patient's address as soon as a suspected case is identified. Public health workers then visit the home to fumigate and place insecticide in standing water within a 50 m radius of the home. During this time, the location of the home can be geo-referenced. All authors have approved the visualization of exact dengue case locations.

### Determining Larval or Other Risk Indices

Public health workers in Nicaragua perform four cycles of entomologic surveillance for *Aedes aegypti *every year. Each cycle lasts for three months. During these cycles, the municipality is first surveyed for levels of infestation and then resources are directed and interventions are employed depending on the results of the entomologic survey. During their routine epidemiologic surveying, public health workers in Bluefields collected data to calculate neighborhood-specific indices of larval infestation and density: the home index (discussed below), the Breteau index, the deposit index, and the neighborhood block index [12]. Neighborhoods were color coded to indicate a number of parameters, including levels of infestation, access to running water, or population density. Levels of infestation were measured by public health surveyors.

Ten percent of all of the homes in within each neighborhood block were surveyed for any sources of standing water. Surveillance of ten percent is dictated by the Nicaraguan Ministry of Health in order to provide an estimate of the level of infestation per municipality [12]. While surveying more than ten percent of the homes would provide a greater level of precision, surveillance is time consuming. For example, the municipality requires one month to survey ten percent of Bluefields, leaving only two months for interventions. In order to survey ten percent of Bluefields, the number of houses in each neighborhood block was compiled and then multiplied by 0.10 and rounded to the nearest integer. This value represented the number of houses that were surveyed on that neighborhood block. Public health workers started surveillance at a home and then counted ten houses down for the next house, making sure to start at a different house during each cycle such that the same houses were not continually surveyed.

When a source was identified, it was inspected for mosquito larvae. If larvae were found, the larvae were collected in an ethanol solution and all larvae were reviewed by an entomologist at the Ministry of Health to identify the species of the mosquito larvae. If the larvae were found to be *Aedes aegypti *larvae, then these homes were documented as positive for dengue larvae and added into the calculation of the home index for dengue larval infestation. The home index is calculated by summing all the homes with *Aedes aegypti *larvae discovered divided by the total number of homes surveyed multiplied by one hundred.

According to the risk levels set by the Nicaraguan Ministry of Health, optimal control is a home index of <1%, good control is a home index of <5%, alarm levels of infestation are indicated by a home index of <10%, and emergency levels of infestation are indicated by a home index of 10% or greater.

Access to running water was determined by surveying the home owners at ten percent of the homes in Bluefields to determine if the national water company, Enacal, had pipes running access to water to their neighborhoods and if so, how many hours per day they could rely on flow of the water.

Population density was calculated using the Ministry of Health standard that the average home in Bluefields houses six persons. This number was then multiplied by the number of homes per neighborhood.

In ArcMap, these indices were entered into a table of one the of map layers that describes the attributes of individual neighborhood blocks and these neighborhood blocks were color-coded, with graduating intensity of color representing areas with higher larval indices. In addition, public health workers collected information on the locations of sites predisposed to larval development and the locations of the homes of dengue patients. Examples of sites predisposed to larval development included garbage dumps, areas without running water, collections of standing water, and homes positive for mosquito larva. Reference points such as churches, schools, and commercial buildings were also recorded because patients describe their addresses based on landmarks so such reference point documentation may aid public health workers in localizing patient homes for future interventions.

Finally, points representing likely larval development sites can be overlaid. Such a map conveys the spatial relationship between the specific likely larval development sites and the presence of dengue cases.

## Results

**Figure 1:**
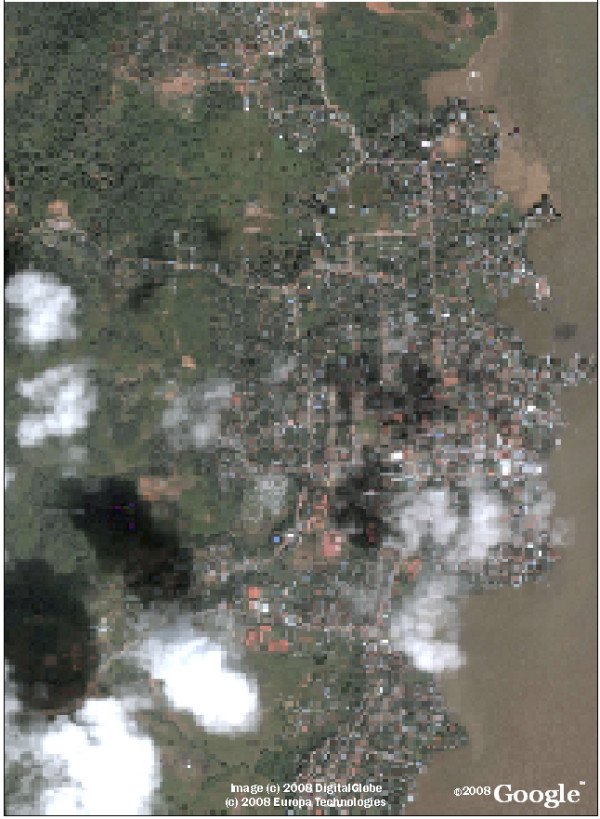
**Bluefields, Nicaragua: Google Earth Satellite Imagery**. Jpeg images from Google Earth were compiled as a mosaic to create this base map to be use in the high performance mapping program ArcGIS.

**Figure 2:**
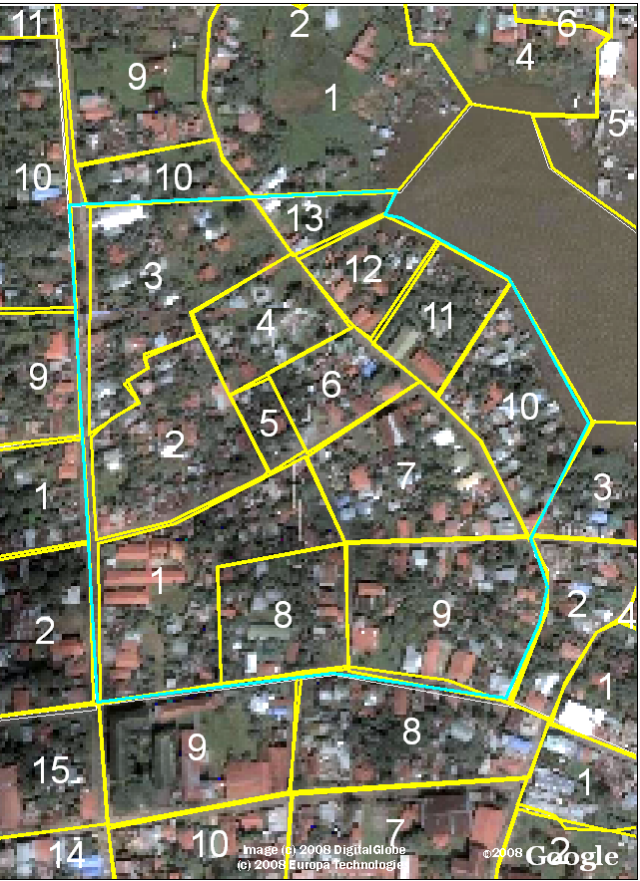
**Example of Printed Satellite Map for Data Collection in Neighborhood Beholden**. Public health workers mark maps like this printed in black and white on 8.5 × 11 inch paper to denote the location of potential larval development sites during their routine epidemiologic surveillance. Each neighborhood block is denoted with its corresponding number.

We created a geo-referenced satellite base map of Bluefields using satellite imagery freely available from Google Earth, as described in the Methods section and shown in Figure 1. The map shows resolution of individual houses, with only certain areas obscured due to cloud cover. This base map was easily manipulated using ArcGIS and Erdas Imagine software without requiring further Internet access and was utilized as the starting point for further analysis and for the creation of the printed neighborhood maps; an example of the Beholden neighborhood is shown in Figure 2.

**Figure 3:**
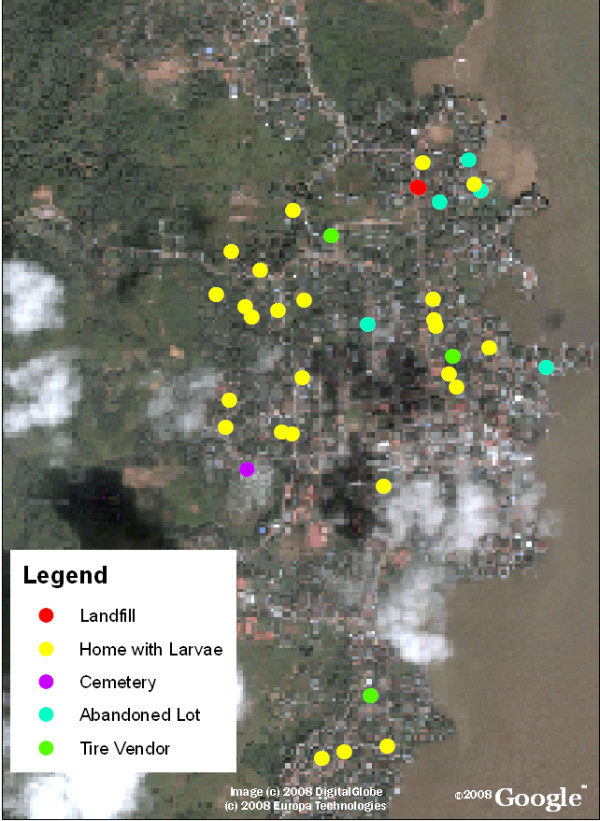
**Bluefields, Nicaragua with the locations of potential larval development sites overlaid**. Dengue risk factors such as abandoned lots, cemeteries, landfills, and garbage dumps that act as locations for mosquito breeding and disease transmission are noted by different symbols on the map.

**Figure 4:**
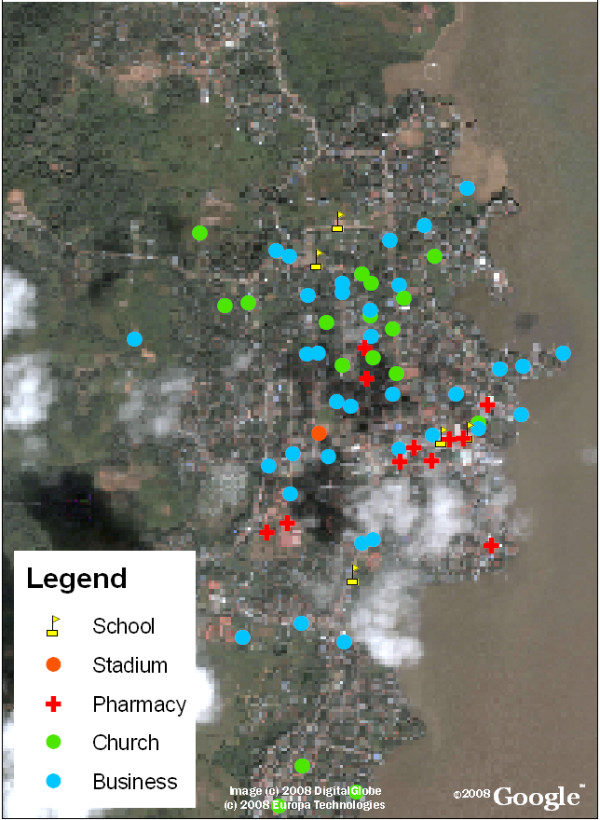
**Bluefields, Nicaragua with the locations of reference points overlaid**. Reference points can be helpful in areas with unplanned urbanization to localize patient homes as the address systems may be underdeveloped. In Nicaragua, addresses are giving based on landmarks such as "the home two blocks towards the lake from the church" so we found it useful to collect information of common reference points in Bluefields.

**Figure 5:**
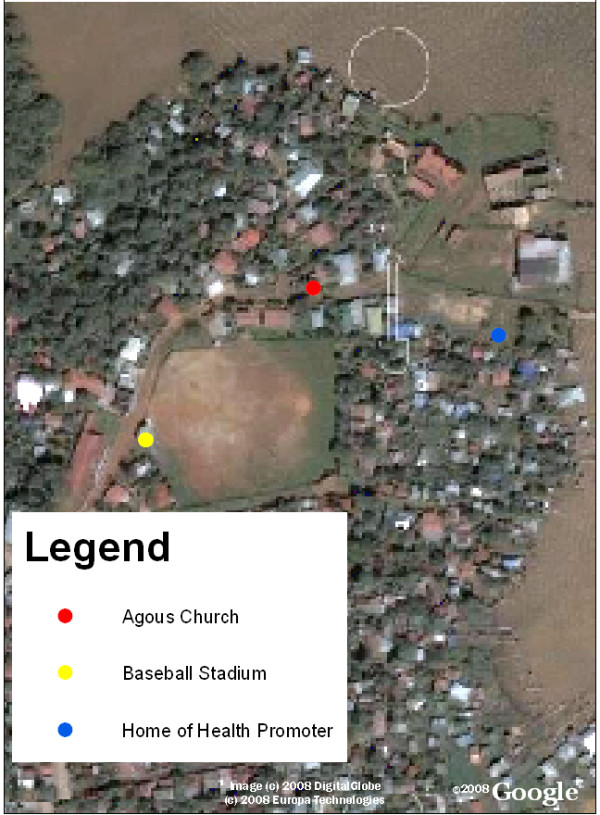
**Bluefields, Nicaragua close-up of reference points**. Here the baseball field, a church, and the home of a health promoter can be visualized.

**Figure 6:**
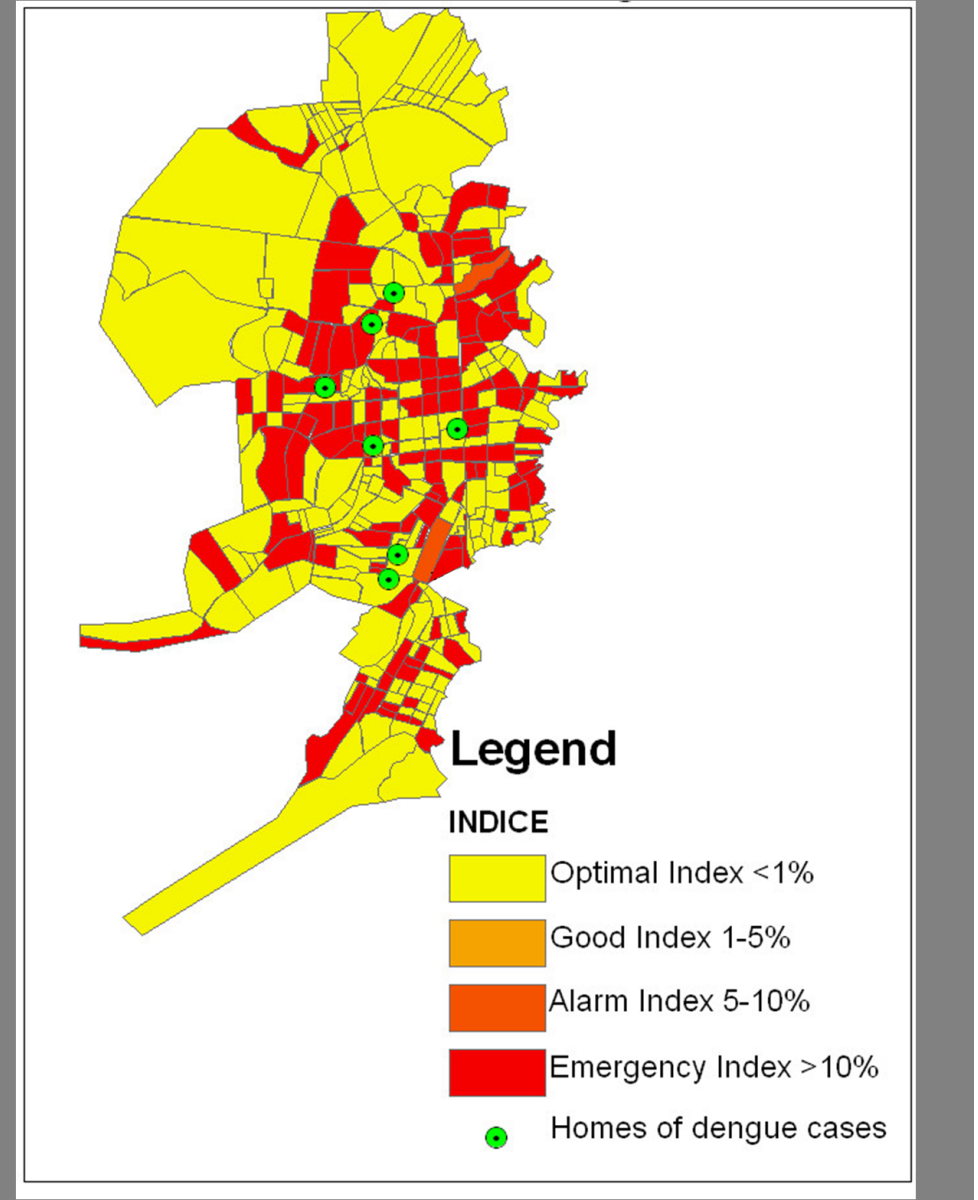
**Bluefields, Nicaragua with Suspected Cases from January 2009 overlaid on indices of infestation by neighborhood block**. Suspected cases of dengue are cases with clinical symptoms of dengue that are awaiting serologic confirmation. Yellow areas indicate optimal control, with home indices less than 1%, light orange areas indicate good control, with home indices between 1–5%, dark orange areas indicate alarm levels with indices of 5–10%, and red areas indicate emergency levels with home indices greater than 10%. The cases are shown in green and generally correlate spatially with the locations of high indices of infestation.

Data collected from the public health surveyors was then entered into the map as described above and utilized to calculate several indices of larval infestation (home index, Breteau index, deposit index, and neighborhood block index). Figure 3 provides further detail of information on locations containing features predisposed to larval development, such as the presence of cemeteries, landfills, homes positive for larvae, and abandoned lots, while Figures 4 and 5 detail significant reference points (such as churches or schools) in Bluefields and its neighborhoods. Known and suspected dengue cases were also entered into the base map using ArcGIS. Figure 6 shows these cases overlaid onto the satellite base map with color-coded indices of infestation denoted by neighborhood.

Once information on the locations of larval development sites and dengue cases had been input on the map using the ArcGIS software, spatial patterns among the known dengue cases, mosquito larval indices, and the previously identified larval development sites were visually analyzed for patterns. The indices we examined were the home index, container index, Breteau index, and neighborhood block index [12]. Figure 6 illustrates that certain neighborhoods have a high home index of infestation. The locations of the homes of cases are overlaid such that one can visually identify areas with high infestation and dengue cases. These areas tended to be neighborhoods with greater population density and significant unplanned urbanization leading to problems with water management. Using this spatial information, the Bluefields public health department was able to prioritize limited supplies of larvicide, insecticide, and human resources to the exact areas documented with the highest indices of infestation. In addition, targeted control interventions were planned to directly address larval development sites in high index neighborhoods based upon the data collected.

## Discussion

We have described the creation of an easy to implement system for creating spatial maps of dengue cases and the location of sites with predilection for larval development that uses the GIS mapping program ArcGIS available from the Global Fund. This can allow deployment of focused interventions in an efficient and cost-effective manner. Larval development sites and dengue cases could be marked either with GPS units when available or on printed maps generated from the satellite base map and then entered into ArcGIS for further analysis. Both methods yielded data point accuracy within 5–10 m, though the printed map method could only be utilized for those areas where ground features were not obscured by cloud cover or shadows.

To create the base maps, we utilized images from Google Earth. This is a useful resource for base-map creation because the imagery is open access and can be easily captured and manipulated. Google Earth images are readily available for use without cost for such scholarly and not for profit purposes (including educational activities or scholarly publication) through the 'fair use' clause of the Google permission guidelines, provided that appropriate attribution is given by reprinting the copyright attribution text and Google logo [13]. Google was also notified of the use of imagery for this purpose.

An important consideration is resolution of the satellite imagery. As the *Aedes aegypti *mosquito has a dispersal of approximately 50 m when released in an urban environment, it is useful to identify the likely indoor or closed-in breeding sites and range within which intervention is necessary, which in this case was within 50 m of a known dengue case or identified larval breeding area. The aerial maps in Google Earth had sufficient resolution to permit accurate mapping of structures within this radius. Although Google Earth has excellent resolution in Bluefields, cloud cover obscures the aerial view in some neighborhoods. To address the problem of cloud cover in Bluefields, public health workers used the track function on the GPS devices to geo-reference streets and the point function to note homes that appeared obscured in the aerial views. This information was then laid over the cloud cover in ArcGIS to functionally complete the map in areas where the image was obscured. Also, map resolution varies between different areas of the country or different parts of the world. Cloud cover and resolution was not a significant problem in the images of Bluefields but this use of Google Earth imagery may not be possible in certain areas of the world due to poor resolution, especially if tracking vectors with a shorter range than *Aedes aegypti*. Fortunately, dengue fever is a primarily urban disease and Google Earth tends to have better resolution of urban areas [9].

Another consideration is that Google Earth imagery is not updated in real-time and the imagery may not represent new buildings or other developments placed after the capture of the images. Therefore, it may be useful to record the date when the imagery was captured and update the map when new imagery is available.

The production of this map required the additional steps of image screen capture, formation of a digital mosaic of the images, and georeferencing of the images with handheld GPS units. These steps could potentially be avoided if georeferenced images were already available (possibly as a donation from imagery providers) or possibly by writing or obtaining software to screen capture the whole image. However, should such georeferenced images or the screen capture software not be available, these steps are one option to allow for georeferencing and integration of available satellite images. Additionally, this method of constructing a base map can be employed using any type of digital imagery that an organization may have available, such as aerial photos. For example, this system may be used in the near future to georeference aerial photos of El Bluff, Nicaragua, which is a tiny island off the coast of Bluefields not picked up on Google Earth imagery but is a site of a significant number of dengue infections. Another benefit of performing one's own georeferencing is that the data point accuracy can be improved to a positional accuracy of 5–6 m. Google Earth has a positional accuracy of 39.7 m (root-mean-squared error). Developed countries have an improved root-mean-squared error of 24.1 m, however in developing countries the control points are significantly less accurate, particularly away from urban areas [14]. Therefore, in disease processes such as dengue where the vector is commonly limited to a 50 m radius or less, proper control may require creation of maps with greater data point accuracy than available through Google Earth, which can be developed using this process.

In this analysis, visual inspection was utilized to identify areas with dengue cases and high indices of infestation that require prioritized interventions. Visual inspection is intuitive and the maps provided administrators at the Ministry of Health sufficient information to target interventions. Further spatial analysis can easily be performed to provide more information to guide interventions if necessary or as desired. For example, ArcGIS readily provides multiple statistical tools including cluster analysis and tools for measuring geographic distributions. Cluster analysis may be useful for further identification of locations with groups of high indices or multiple cases. Tools are also available that allow one to identify a central point around which cases are distributed and the location of the most central case reported.

In addition, if an investigator would like to perform a more detailed spatial analysis of cases with statistics not available through the selected GIS software, a commonly used statistic for spatial temporal cluster analysis is the Knox test [15,16]. While these statistical methods can provide additional information, we found that visual inspection provided adequate information to guide interventions such as fumigation and insecticide distribution.

Another consideration for the design of the mapping system was that in Nicaragua, few municipalities have access to Internet so it was critical that this system did not depend on this modality beyond the creation of the initial complete mosaic map at the central Ministry of Health. The final system delivered to the municipality was a series of saved images that could be transferred on CD and manipulated in ArcGIS. Thus, Internet access is not needed for the continued functioning of the mapping system.

One advantage of working in ArcGIS rather than Google Earth for the analysis of the maps is that ArcGIS is a program better suited for epidemiologic mapping and interpretation. With a few simple commands in ArcGIS, GPS input is represented as a shapefile, neighborhood blocks can be color-coded to indicate stratified levels of infestation, and statistics can be performed. In addition, there are a myriad of other functions that can be performed using ArcGIS in the analysis of disease patterns. Furthermore, much of the infrastructure for the mapping of dengue is already in place thanks to a five year project from 2000–2005 by the Global Fund for AIDS, Tuberculosis, and Malaria. This organization funds proposals to strengthen health systems in 123 developing and transitional countries worldwide. In Nicaragua, the Global Fund has been instrumental in setting up GIS for malaria surveillance, which included purchase of equipment such as ArcGIS, GPS devices, computers, and human resources development [17]. Thus, technology training and devices implemented by the Global Fund to control malaria may also be used for dengue control with minimal additional cost.

While ArcGIS can be an appropriate platform for mapping in developing countries that have already purchased the program and trained public health administrators in its use, it would be cost-prohibitive in any setting where the program was not already purchased or available through a mechanism such as the Global Fund as the program costs hundreds of dollars per license. There are numerous free or low-cost alternatives to high-end ArcGIS software that should be considered for the future of mapping in resource-limited settings [18]. These include SaTScan, a program that can be used to perform geographical surveillance of disease using space-time clustering [19], MapServer, an open-source platform for publishing spatial data and interactive mapping applications [20], and Quantum GIS, which can perform spatial analysis of shapefiles [21] These open source Web GIS software systems now have the sophistication, stability, and user-friendliness to compete with commercial GIS products such as ArcGIS and can be utilized to create maps similar to ones described in this article; they will be particularly important for use in resource limited settings [18].

Nonetheless, the present use of ArcGIS already available to many developing countries through the Global Fund combined with Google Earth represents a drastic improvement from practices in the recent past. Previously, in Bluefields, administrators were forced to guide interventions based solely on a table of the indices of infestation per entire neighborhood. Now, using aerial mapping of cases and likely larval development sites, high indices are color-coded and statistically analyzed per neighborhood block leading to greater spatial accuracy, in addition to precisely mapping larval development sites, homes of patients, and reference points. In addition, municipalities on the Atlantic coast often have difficulties securing funds from the central Ministry of Health for resources such as insecticide, gasoline for fumigation, and funds for contracting workers as it is difficult to communicate the extent of work being performed to Managua. By emailing maps of the spatial progress of intervention efforts in addition to resource costs, administrators in Managua are better able understand what resources are needed.

Although the Ministry of Health has record of the approximate address of the homes of dengue cases it is important to map other locations of larval development as people often travel from their homes for work. Thus this mapping system represents a significant increase in the amount of information collected and the detail of its presentation, which can then be utilized to design and implement specifically targeted interventions, ultimately saving time and money.

**Figure 7:**
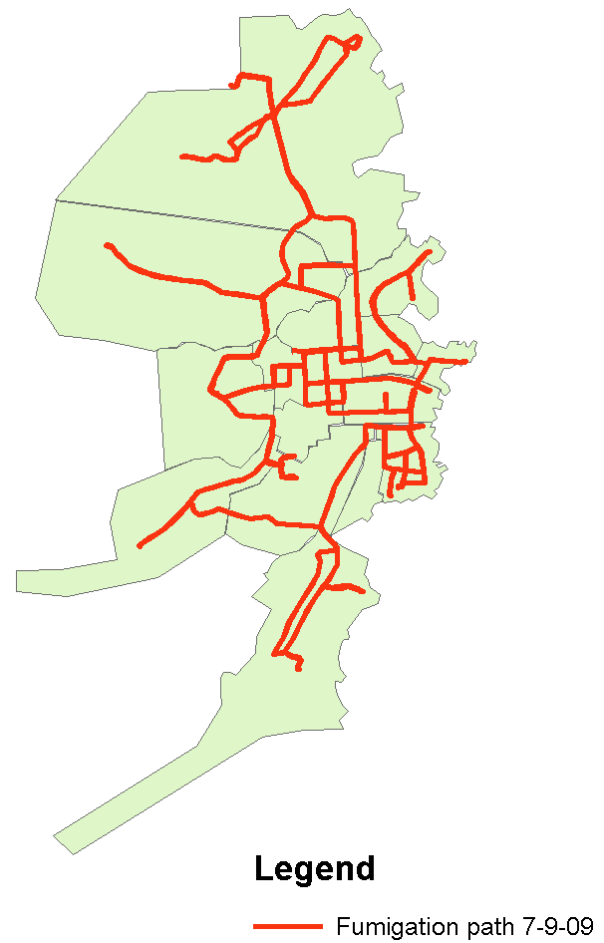
**Emergency Spatial Fumigation performed on 7/9/09 during a Dengue Epidemic**. This map was constructed using the track function on the GPS unit to collect information on the path taken by the fumigation truck as it drove through areas of Bluefields. The map was then sent to the Ministry of Health in Managua to communicate the activities of the day and plan for control interventions for the following day. This map also demonstrates areas that cannot be reached by truck as the streets are two narrow. These areas are of particular risk as both the fumigation truck cannot enter and the garbage truck does not enter. Therefore, further resources are needed to send public health workers into these neighborhoods to fumigate with handheld devices, to clean up garbage, and to educate people about the importance of carrying their garbage out to a site where the garbage can be properly disposed of.

Currently, this system is being used to map a recent dengue outbreak in Bluefields with 125 confirmed cases and approximately 600 suspected cases of dengue. In addition to demonstrating the location of dengue cases, mapping of intervention efforts has been important in communicating resource needs to the central Ministry of Health. Mapping of the area fumigated daily using gasoline and cipermetrina allows administrators at the central level in Managua to recognize the extent of the interventions being employed on a daily basis such that plans can be developed and resources sent accordingly. Figure 7 demonstrates the map of the pathway of a truck performing emergency spatial fumigation during a dengue epidemic that was sent to Managua to communicate intervention activities as a justification for further resource allocation.

The base maps created through the system described above are not limited to analysis of dengue but have broad potential uses for many diverse public health applications. For instance, this system is currently being used by the Ministry of Health to map the locations of pharmacies in an effort to elucidate deficiencies in access to medications for patients in Bluefields. In addition, the Ministry of Health has plans to partner with Enacal, the national water company, to map access to potable water to prevent a wide range of water-borne diseases. The system is also being used to map malaria, leishmaniasis, and recently influenza cases in Bluefields. Therefore, this mapping program is not only useful for directing resources for dengue but can be applied towards the management of multiple diverse public health issues.

## Conclusion

The mapping system described above can be employed as a low-cost management tool for the control of dengue in many developing countries as it does not depend on continuous Internet access to function, and the GIS software is available to developing countries through the Global Fund. The availability of high quality satellite maps from Google Earth and the use of this GIS software make the system accessible at minimal cost. While GPS units are ideal for localizing cases and larval development sites, they are not necessary, and the use of printed maps is a low-cost solution that can easily be utilized for localizing likely larval development sites in order to reduce the incidence and burden of dengue of similar transmissible diseases. The availability of these epidemiological tools will allow for interventions to be targeted specifically to areas of greatest need, conserving scarce resources towards the goal of reducing the burden of disease.

## Abbreviations

DF: Dengue Fever; DHF: Dengue Hemorrhagic Fever; GIS: Geographic Information System; GPS: Global Positioning System; SILAIS: Local System for the Integral Attention to Health.

## Competing interests

The authors declare that they have no competing interests.

## Authors' contributions

AYC conceived the program with the creation of the base-map, creation of the study design, implementation of the mapping project, analysis of the data, and drafting of the manuscript. RPK contributed to the design and coordination of the study, in addition to the analysis of data and drafting of the manuscript. MEP participated in the creation of the base-map, creation of the study design, implementation of the mapping project, and analysis of the data. JJ contributed to the design and coordination of the study, secured the implementation of the mapping project, and performed analysis of data. MES contributed to the study design and revision of the manuscript. SMH contributed to the study design and revision of the manuscript. DJC assisted with data collection and coordination with MINSA. All authors have read and approved the final manuscript.
